# Compound‐complex odontoma: A rare case report

**DOI:** 10.1002/ccr3.5658

**Published:** 2022-04-04

**Authors:** Chaima Khalifa, Mounir Omami, Maroua Garma, Afef Slim, Sameh Sioud, Jamil Selmi

**Affiliations:** ^1^ Department of Oral Medicine and Oral Surgery Dental Clinic of Monastir Monastir Tunisia; ^2^ Faculty of Dental Medicine Laboratory of oral health and maxillofacial rehabilitation (LR12ES11) University of Monastir Monastir Tunisia

**Keywords:** compound‐complex odontoma, odontogenic tumor, odontoma, radio‐opacities

## Abstract

The World Health Organization (WHO) classifies odontomas as odontogenic tumors, consisting of odontogenic epithelium and ectomesenchyme. They result from developmental abnormalities and, therefore, do not constitute authentic tumors. It is a local malformation that has no growth autonomy. They are rarely symptomatic and are usually discovered accidentally during the realization of a radiographic examination. There are two variants of odontomas: complex and compound. Complex odontomas are made of a mass consisting of an anarchic assembly of mineralized tissue (enamel, dentin, and cementum) and dental pulp; while compound odontomas are consisting of a set of small rudimentary teeth, assembling in clusters. They rarely show the features of both types together. The aim of this work is to report a rare presentation of an odontoma in a 24‐year‐old male patient, which present the characteristics of both complex and compound variants. Surgical excision of the lesion was performed. Anatomopathological examination confirmed the diagnosis. Clinical and radiological survey does not show any recurrence.

## Abstract 2

Compound‐complex odontoma is an uncommon variant of odontoma; it is detected through routine radiographic examination. It is, therefore, advisable to conduct an accurate diagnosis in order to avoid subsequent complications. It is recommended to perform histopathological analyses so as to discard the possibility of association to some other type of odontogenic tumors.

## 1 INTRODUCTION

Odontomas are the most prevalent odontogenic tumors of the jaws in an interval of 35%–76%, characterized by their non‐aggressive character.^1^ Their discovery is usually accidental during routine radiological examinations given their asymptomatic evolution in most cases.^2^ At the beginning of their discovery, odontomas were considered true tumors. But very quickly, this appellation disappeared to be replaced by the notion of hamartomas malformations as they develop from odontogenic epithelium and ectomesenchyme components, with the capacity of forming enamel, dentin, and cementum.^3^ The etiopathogenesis of these tumors is unknown. However, trauma in primary dentition, periodontal Malassez remains, inflammatory processes, odontoblastic hyperactivity, and hereditary anomalies are considered as possible etiological factors.^2^ The World Health Organization (WHO) classified odontomas into two variants: compound and complex.^4^ The compound form consists of all the tissue structures involved in the formation of the teeth; these different tissue structures can be associated with each other to form a variable number of tooth‐like structures, called “odontoids.” Although, a complex odontoma is a malformation in which all dental tissues are represented and arranged in an anarchic way.^4^ The complex odontomas appear to be more common than composite odontomas.^3^ The distribution between sexes is approximately equal, and the average age of the patients is between 20 and 30 years in most studies.^5^ Complex odontomas occur commonly in posterior region of the mandible, while compound odontomas occur principally in the anterior part of the maxilla.^3^ However, odontomas rarely show both radiological and histological features of compound and complex types together and this type of odontoma is known as compound‐complex odontoma in the literature.^5^

We aimed to present a rare case of a large compound‐ complex odontoma, occurring in the mandible in a young patient. It would have a pedagogical and epidemiological interest as it shows a clear example of an uncommon variant of odontoma, which exhibits the characteristics of both composite and complex types.

## 2 CASE REPORT

**FIGURE 1:**
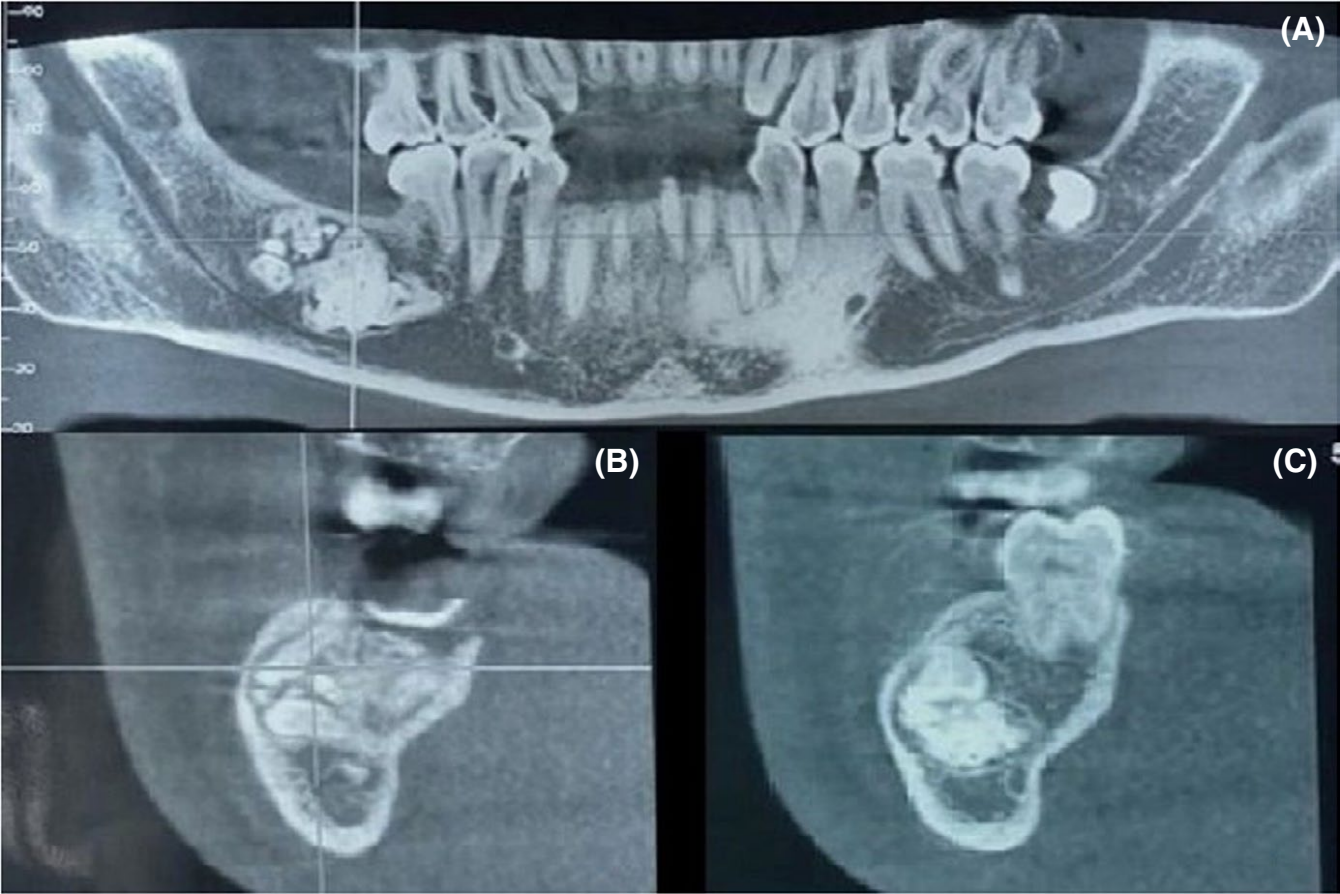
Cone Beam Computed Tomography (CBCT) findings of radio‐opacities in the right posterior region of the mandible. (A) CBCT panoramic reconstruction showing the extension of the lesion. (B) Coronal CBCT view showing expansion of the buccal bone cortical and an assemblage of teeth‐like structures. (C) Coronal CBCT view showing an anarchic radiopaque image

**FIGURE 2:**
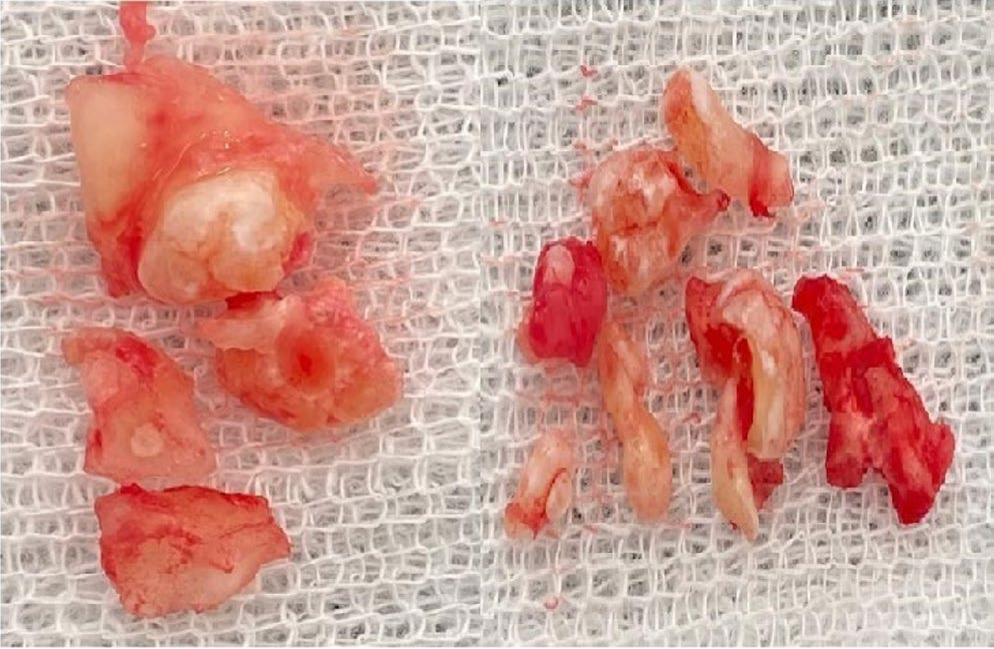
The excised specimen composed of calcified part and teeth‐like structures

A 24‐year‐old male patient presented to the Department of oral medicine oral surgery of the university dental clinic of Monastir, Tunisia with the chief complaint of pain in mandibular right wisdom teeth. Patient's medical and family histories were non‐contributory. The clinical examination showed, in addition to insufficient hygiene, a disorder of the mandibular anterior teeth, the right mandibular wisdom tooth was decayed. A routine panoramic radiograph showed a large radiopaque lesion in the right posterior mandible delimited by a radiolucent halo and extended from the first molar to the second molar region. The lesion was composed by two parts. The first one consisted of an assemblage of multiple odontoids and the second one composed of an anarchic radiopaque image. The cone‐beam computed tomography revealed a lesion involving the right body of mandible and shifted more on the buccal side. There was an expansion and thinning of the buccal cortical of the mandible (Figure 1). According to these findings, provisional diagnosis of odontoma was considered. The surgical treatment carried was an extraction of the right wisdom teeth and surgical excision of the lesion by curettage (Figure 2). The excised specimen was fixed and sent for anatomopathological study. Based on the macroscopic clinical image of the resected tissue and histopathological examination, the diagnosis of compound‐complex odontoma had been confirmed. Six months follow‐up showed no relevant recurrence.

## 3 DISCUSSION

Odontomas represent an important entity among maxillary benign odontogenic tumors.^3^ Patients often have no pain, and odontoma is accidentally diagnosed during routine checks or during a delayed eruption of permanent teeth or, more rarely, of milk teeth.^6^ Odontogenic tumors may occur at any period of life from epithelial tissue, ectomesenchyme, or both with or without hard tissue formation.^7^ The etiology of odontomas is uncertain. There are several hypotheses: local trauma during primary dentition, infection, family history, hereditary abnormality, odontoblastic hyperactivity, or spontaneous genetic mutation.^8^ Compound odontoma, as well as complex odontoma are benign, slow‐growing pathological entities and self‐limiting.^9^ Despite the high incidence of this tumor, compound‐complex type, which shows both radiologic and histologic characteristics of compound and complex types is not common in the literature.

A bibliographic research on PubMed platform was performed. The articles included were written in English language and published until 2021. Only five publications (five case reports and one retrospective study) report cases of compound‐complex odontomas.^5^, ^10^, ^11^ Therefore, it shows that our case report is uncommon.

There are different clinical aspects of odontoma. It can be either intra‐osseous, extra‐osseous or erupted.^5^ Most cases are intraosseous as seen in our case. Although the anterior region of the maxilla (67%) is the most prevalent location,^12^ our case was reported on the posterior region of the mandible. Radiological appearance may vary depending on the stage of development and degree of mineralization of the tumor. Odontomas may be seen as radio transparent in the beginning. Increasing mineralization leads to a corresponding increase in calcifications, with constant decrease in radiotransparence. Mature complex odontomas appear under the form of round or ovoid radio‐opacities with well‐defined edges. The compound odontoma consists as a number of radio‐opacities of different sizes, which resemble small dental structures.^13^ On histopathological examination, these two forms of odontomas consist of all hard dental tissues—enamel, dentin, and cementum—as well as pulp tissue. These different tissues are mixed anarchically in complex odontoma, whereas in the compound odontoma, they organize themselves like a natural tooth, which is clearly recognizable. Both types of lesions are surrounded by a fine conjunctive capsule.^5^

Differential diagnosis must be performed with ameloblastic fibroma, ameloblastic fibro‐odontoma, and odontoameloblastoma.^13^

Odontomas can also manifest as part of syndromas, such as Gardner syndrome, basal cell nevus syndrome, familial colonic adenomatosis, Tangier disease, or Hermann syndrome.^3^ Such association was not seen in the presented case.

The treatment consists in a surgical removal of the lesion with curettage. Their enucleation is simple as they are capsulated tumors.^2^ The recurrence is not common.^12^ But, an early discovery and management allows us to be more conservative during the surgery, to avoid degeneration of the lesion and retain the vitality and the placement of adjacent tooth and eventually ensure a good prognosis.

## CONFLICT OF INTEREST

The authors declare that they have no conflict of interest or sources of funding for this particular study.

## AUTHOR CONTRIBUTIONS

Khalifa Chaima has written the manuscript. Omami Mounir has participated in the design, the acquisition of data as well as the revision of the manuscript. The other authors discussed the results by revising critically for important intellectual content and have given final approval of the version to be published. All authors approved the final draft of the manuscript.

## ETHICAL APPROVAL

Our institution does not require ethical approval for reporting individual cases or case series.

## CONSENT

Written informed consent was obtained from the patient for his anonymized information to be published in this article.

## Data Availability

The datasets generated during the current study are not publicly available but are available from the corresponding author on reasonable request.

## References

[1] Levi‐Duque F , Ardila CM . Association between odontoma size, age and gender: multivariate analysis of retrospective data. J Clin Exp Dent. 2019;11(8):e701‐e706. 31598198 10.4317/jced.55733 PMC6776404

[2] Abdul M , Pragati K , Yusuf C . Compound composite odontoma and its management. Case Rep Dent. 2014;2014:107089. 25587458 10.1155/2014/107089 PMC4283421

[3] Hidalgo‐Sánchez O , Leco‐Berrocal MI , Martínez‐González JM . Metaanalysis of the epidemiology and clinical manifestations of odontomas. Med Oral Patol Oral Cir Bucal. 2008;13(11):E730‐E734. 18978716

[4] Soluk‐Tekkeşin M , Wright JM . The world health organization classification of odontogenic lesions: a summary of the changes of the (4th) edition. Turk Patoloji Derg. 2017;2018:34. 10.5146/tjpath.2017.01410 28984343

[5] Soluk Tekkesin M , Pehlivan S , Olgac V , Aksakallı N , Alatli C . Clinical and histopathological investigation of odontomas: review of the literature and presentation of 160 cases. J Oral Maxillofac Surg. 2012;70(6):1358‐1361. 21840103 10.1016/j.joms.2011.05.024

[6] Isola G , Cicciù M , Fiorillo L , Matarese G . Association between odontoma and impacted teeth. J Craniofac Surg. 2017;28(3):755‐758. 28468159 10.1097/SCS.0000000000003433

[7] ‐ Wright JM , Soluk Tekkeşin M . Odontogenic tumors: where are we in 2017? J Istanb Univ Fac Dent. 2017;51(3 Suppl 1):S10‐S30. Published 2017 Dec 2. 29354306 10.17096/jiufd.52886 PMC5750825

[8] Yadav M , Godge P , Meghana SM , Kulkarni SR . Compound odontoma. Contemp Clin Dent. 2012;3(Suppl 1):S13‐S15. 22629054 10.4103/0976-237X.95095 PMC3354782

[9] Boffano P , Zavattero E , Roccia F , Gallesio C . Complex and compound odontomas. J Craniofac Surg. 2012;23(3):685‐688. 22565876 10.1097/SCS.0b013e31824dba1f

[10] Smith GC . An interesting presentation of a complex‐compound odontome. Aust Dent J. 1985;30(4):265‐267. 3866527 10.1111/j.1834-7819.1985.tb02506.x

[11] Samuels HS , Gerry RG . Complex‐compound odontoma: report of case. J Oral Surg Anesth Hosp Dent Serv. 1963;21:348‐352. 13976167

[12] Chang JY , Wang JT , Wang YP , et al. Odontoma: a clinicopathologic study of 81 cases. J Formos Med Assoc. 2003;2013:876‐882. 14976568

[13] Gedik R , Müftüoğlu S . Compound odontoma: differential diagnosis and review of the literature. West Indian Med J. 2014;63(7):793‐795. 25867569 10.7727/wimj.2013.272 PMC4668987

